# Clinical Experience with Tigecycline in the Treatment of Prosthetic Joint Infections

**DOI:** 10.7150/jbji.34866

**Published:** 2019-05-21

**Authors:** Allison Lastinger, Nathanael McLeod, Matthew J Dietz, John Guilfoose, Arif R Sarwari

**Affiliations:** 1 Department of Medicine, West Virginia University;; 2 Department of Orthopaedics, West Virginia University.

**Keywords:** Tigecycline, Prosthetic joint infection, Biofilm infection, Arthroplasty infection

## Abstract

*Purpose:* The purpose of this study was to examine the use of tigecycline in the treatment of prosthetic joint infection (PJI).

*Methods*: This is a retrospective review performed from 2008 to 2017, examining adult patients with PJI at a tertiary medical referral center who received tigecycline for 75% or greater of the treatment course. Failure was defined as need to return to the operating room for an infectious complication or persistent drainage from the joint.

*Results*: A total of 37 patients met inclusion criteria. The median age was 65 years, and 65% of patients were female. The most common reasons for tigecycline use were culture negative infection, polymicrobial infection, and renal failure, but other reasons included antimicrobial allergies and resistant organisms. The mean duration of tigecycline therapy was 40 days (range 28-52 days). Treatment success was documented in 16 cases (43%).

*Conclusions*: Tigecycline is a glycylcycline approved for treatment of a variety of infections including skin and soft tissue infections, but little is known about its use in the treatment of PJI. We found that tigecycline is well-tolerated for prolonged durations. Our success rate was 43%, but the majority of patients in this study had complicated infectious surgical histories and had received prior prolonged courses of antimicrobial therapy which likely affected treatment outcome. We concluded that tigecycline should be reserved as an alternative when other antimicrobials for PJI have been exhausted. More studies are needed to assess tigecycline's use in the treatment of PJI.

## Introduction

In 2006, nearly 800,000 total hip and total knee arthroplasties were performed in the United States 1. Prosthetic joint infection (PJI) is the most serious complication, occurring in 0.8 to 1.9% of knee arthroplasties and 0.3 to 1.7% of hip arthroplasties 1. As the population ages, the number of people requiring arthroplasties is expected to increase dramatically 2, 3. Prosthetic joint infections are not only associated with major morbidity but also significant mortality 4, 5.

Treatment of PJI is challenging and typically involves a combination of surgery and a prolonged course of intravenous (IV) antimicrobial therapy. *Staphylococcus aureus* and coagulase-negative staphylococci cause 65% of prosthetic hip and knee infections 6. Up to 20% of cases are polymicrobial, and in 7-11% of cases, no organism is isolated 1, 6. For oxacillin susceptible gram-positive cocci, nafcillin and cefazolin are commonly used, while vancomycin remains the drug of choice for oxacillin resistant gram-positive cocci. Recent studies have focused on antimicrobials that are easier to dose, have fewer toxicities, and have greater efficacy in the setting of biofilm infections. Both linezolid and daptomycin have been studied as potential alternatives to vancomycin with varying success 4.

Treating biofilm infections is challenging, as many traditional antimicrobials are ineffective. Without microcirculation to deliver antimicrobials, it is difficult to penetrate foreign body-associated infections. A recent study suggested that tigecycline might be more effective than other antimicrobials in its ability to treat biofilm infections 7.

To our knowledge, there are only a few case series describing tigecycline's use in the treatment of PJI, each including 3 or 4 patients 8, 9, 10. At our institution, tigecycline is used as a second or third line agent in patients with polymicrobial or culture negative PJI. One of tigecycline's advantages is its broad spectrum of activity. It is effective against gram-positive organisms including methicillin resistant *Staphylococcus aureus* (MRSA) and vancomycin resistant *Enterococcus* species (VRE), many anaerobic organisms, and gram-negative organisms with the exception of *Pseudomonas* and *Proteus* species.

The aim of this study was to perform a retrospective review of our experience with tigecycline in treatment of PJI.

## Methods

A database search was performed to identify all patients at our tertiary referral medical center diagnosed with a PJI based on the 2011 Musculoskeletal Infection Society (MSIS) Criteria and treated with tigecycline from January 1, 2008 to June 1, 2017 11. We retrospectively collected data on demographics, co-morbidities, surgical procedure, pathogen, rationale, duration of tigecycline use, major adverse effects, and clinical outcomes. The study protocol was approved by the Institutional Review Board prior to data collection.

We identified all patients with an International Classification of Diseases (ICD)-9 or ICD-10 code for PJI who received tigecycline from January 1, 2008 to June 1, 2017. We conducted a chart review. Patients were included if 1) tigecycline was prescribed for treatment of PJI (and not another indication); 2) duration of tigecycline therapy was 75% or greater of the total treatment course; 3) follow-up was 12 months or more after surgery. Patients who initially received another antimicrobial regimen and were transitioned to tigecycline were included as long as they received tigecycline for 75% or greater of the treatment course.

All patients were age 18 or older. Demographic data included patient age, sex, weight, body mass index (BMI), and chronic kidney disease stage according to the National Kidney Foundation Kidney Disease Outcomes Quality Initiative 12. We assessed the following co-morbidities: diabetes, renal disease, and immunosuppression. In most cases, therapy was initiated in the hospital and completed in the outpatient setting. The rationale for tigecycline use was determined by review of the Infectious Diseases consult note. We noted the sequence of antimicrobial use for the specific PJI episode under review. If tigecycline was the first antimicrobial used for the episode under review but the patient had received prior courses of antimicrobials for PJI, we noted this. If the patient was started on other antimicrobial therapy initially and then transitioned to tigecycline later in the treatment course, this was also documented. Total duration of antimicrobial therapy for the particular episode was determined including duration on tigecycline.

The patient's entire orthopedic surgical history and microbiologic data were reviewed. We recorded the prosthesis site and type of surgical procedure. Surgical procedures were divided into single-stage revisions, two-stage revisions, 2+ stage revisions, debridement and prosthesis retention, and fusion. Patients in the 2+ category had already undergone an antibiotic spacer, but had evidence of persistent infection. Therefore, they underwent placement of another spacer and received tigecycline during that treatment course. One patient included in the study was managed non-operatively and treated with IV antimicrobials alone. For each surgery, 2 to 8 operating room specimens were sent for culture. Culture data were reviewed, and the resistance profile of the organism(s) was also noted. The infection was defined as 'culture negative' if operating room and joint aspirate cultures for that particular episode did not isolate an organism, but the case still met criteria for diagnosis of PJI by the MSIS definition.

Treatment failure was defined as need to return to the operating room or persistent drainage from the joint. If there was a plan for follow-up surgery at the time of the initial surgery, this was not defined as failure, but any case where a patient required further unplanned surgery was defined as a failure. Follow-up was defined as time elapsed between surgery and the last follow-up visit with Orthopedics or Infectious Diseases. Patients with short follow-up were included if they could be reached by phone for a follow-up survey. We also examined adverse reactions, defined as reactions requiring change in therapy.

## Results

A total of 37 patients with PJI were treated with tigecycline for 75% or greater of their treatment course. This included 24 females and 13 males. The mean age was 65 years (47 to 88 years), and the mean BMI was 35 kg/m^2^ (17.0 to 51.1 kg/m^2^). Eleven patients had Stage 3 or 4 chronic kidney disease, and ten patients were diabetics. See Table 1 for demographic information.

For a more detailed breakdown of individual patient details, please see Table S1.

### Antimicrobial Regimen

The most common rationales for use of tigecycline were renal failure, polymicrobial infection, and culture negative infection. Tigecycline was selected in 14 cases as empiric treatment for culture negative infection and 7 cases due to polymicrobial infection. Five patients had acute kidney injury, while 6 patients had a history of chronic kidney disease. Allergies to other antimicrobial regimens, *Clostridium difficile* colitis, and growth of resistant organisms were additional reasons for tigecycline use. See Table 1. Tigecycline was used as the initial antimicrobial during the specific episode in 32 of the 37 cases (86%), but 20 of those 32 cases (54%) had a history of infection in the joint under review and had received previous courses of antimicrobials during a prior episode. Approximately 59% of all patients (22 of 37) had previously received prolonged courses of IV antimicrobials for prior episodes of PJI in the affected joint.

All patients were treated with the standard dose of tigecycline (100 mg loading dose followed by 50 mg IV every 12 hours). Mean duration of tigecycline therapy was 40 days (range 28 to 52 days). All patients received tigecycline monotherapy except for 3 patients who received fluconazole in addition to tigecycline due to growth of *Candida* species from operating room cultures or yeast on gram stain.

### Microbiology

**Figure 1:**
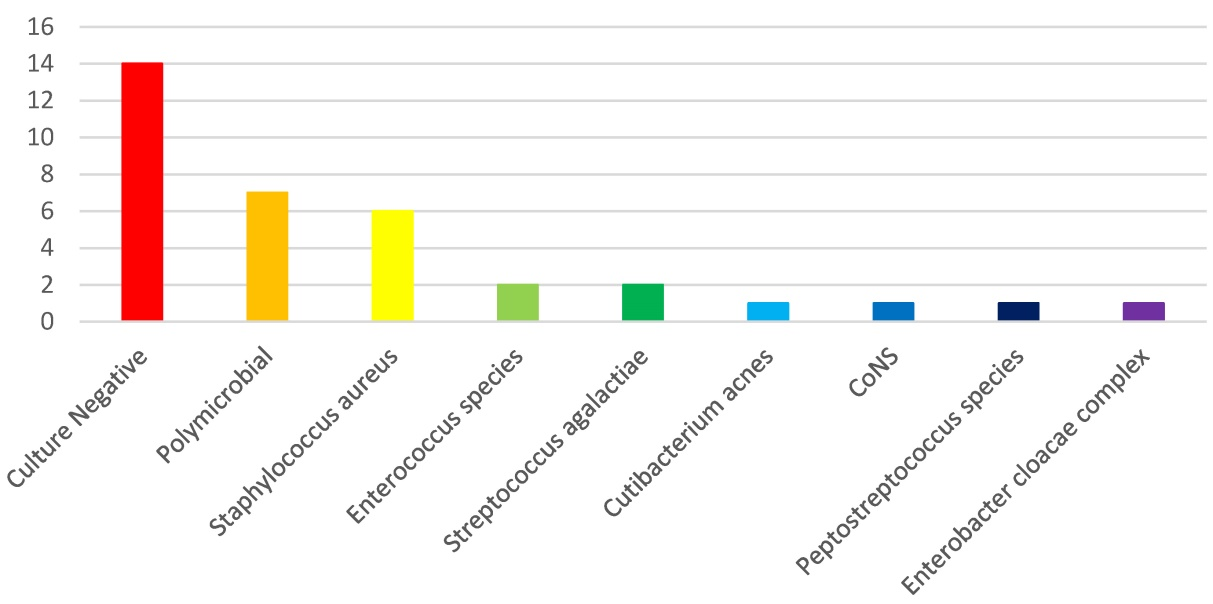
Organisms Isolated from Operating Room Cultures. CoNS, coagulase negative Staphylococcus species.

*Staphylococcus aureus* was the most common microorganism isolated, but a large portion (14 of 37, 38%) of cultures remained negative and these infections were labeled 'culture negative.' See Figure 1. In two of the 'culture negative' cases, the gram stain demonstrated organisms, but ultimately, there was no growth on culture. Seven of the cases were polymicrobial with isolation of more than 1 organism, while 15 of the cases were monomicrobial. Two *Staphylococcus aureus* isolates were resistant to clindamycin, and one methicillin susceptible *Staphylococcus aureus* (MSSA) isolate had a vancomycin MIC of 2 mcg/mL. One *Enterococcus faecium* isolate was resistant to both vancomycin and daptomycin. See Table S2. Interestingly, in two cases, cultures grew *Candida* species and in one case, the gram stain demonstrated budding yeast but ultimately cultures did not isolate an organism. In these 3 cases, the Infectious Diseases service added tigecycline to fluconazole for empiric antibacterial coverage due to concern for polymicrobial infection.

Please see Table S2 for individual patient details.

### Surgical Approach

Most patients underwent two-stage revision or multi-staged revisions with exchange of antibiotic spacers (2+). Five patients were treated with debridement and prosthesis retention, and two patients required fusion. One patient included in the study was managed non-operatively, but had a very complicated history of prior surgeries. Nineteen hip arthroplasties (51%), 15 knee arthroplasties (40%), and 2 shoulder arthroplasties (5%) were performed. One patient underwent both hip and knee arthroplasties. See Table 2.

### Outcome

Mean follow-up was 32 months. Twenty-one cases (57%) were defined as failures. Two cases returned to the OR for dislocation, and all cultures were negative. One patient was managed non-operatively and continued to experience drainage after a course of tigecycline; therefore, the case was categorized as a failure. The majority of failures (14 of 21, 67%) occurred within the first 6 months. At least 7 of the 21 failures (33%) had received prior prolonged courses of antimicrobials for infection in the joint under review. Table 3 outlines the success rate categorized by type of surgical procedure and culture results.

### Adverse Reactions

The most common adverse reaction noted in the chart was nausea and vomiting, but no patients required a change in therapy due to this side effect. One patient developed elevated liver enzymes, prompting discontinuation of therapy.

## Discussion

Tigecycline is a glycylcycline antimicrobial structurally similar to tetracycline. Advantages to use of tigecycline in treatment of PJI include its broad spectrum of activity and its safety in the setting of kidney disease. Disadvantages include its lack of anti-Pseudomonal coverage and its bacteriostatic activity; thus, it should not be used for the treatment of bacteremia in association with PJI. After an initial safety alert was issued in 2010 by the Food and Drug Administration (FDA), a meta-analysis including 10 clinical trials was conducted in September 2013 and demonstrated increased mortality in patients treated with tigecycline at 2.5% (66/2650 patients) compared to 1.8% (48/2628 patients) with other antibiotics 13, 14. This resulted in approval of a Black Boxed Warning for tigecycline cautioning against its use unless alternatives were exhausted. In our study, tigecycline was used in patients with renal disease, infection with resistant organisms, and multiple antimicrobial allergies; therefore, alternatives were exhausted in most cases.

*In vitro* and animal studies suggest that tigecycline may be useful in the setting of PJI. Vaudaux et al demonstrated that tigecycline is equally effective compared to vancomycin in treatment of MRSA foreign body infection in the rat model 15, while Corvec et al suggested that tigecycline functions better when given in combination with colistin or fosfomycin against extended-spectrum-β-lactamase-producing *Escherichia coli* in a tissue cage model of infection 16. Molina-Manso et al examined *in vitro* susceptibility of staphylococci to antimicrobials in orthopedic biofilm infections 7. They demonstrated that rifampin and tigecycline were the most active antimicrobials in treatment of *Staphylococcus epidermidis* biofilm compared to vancomycin, ciprofloxacin, cloxacillin, and daptomycin.

To our knowledge, there are very few studies examining tigecycline's use in the treatment of PJI. De Sanctis described 3 patients with polymicrobial infections including growth of carbapenem-resistant *Klebsiella pneumoniae* 8. Two patients received monotherapy while the third received tigecycline plus amikacin followed by colistin. Outcome was poor with 2 deaths and the other patient requiring limb amputation. Vila et al reported a series of 3 patients treated for multi-drug resistant *Acinetobacter baumannii* early PJI with combination high dose tigecycline (100 mg every 12 hours) plus a mean 8.7 days of colistin 9. All patients required at least one additional debridement but remained asymptomatic after a median of 2.5 years. Finally, Asseray et al reported 4 patients with multi-drug resistant *Staphylococcus epidermidis* treated with combination tigecycline and either fosfomycin or linezolid with a success rate of 75% 10.

At our institution, tigecycline is typically used as a second or third line drug for the treatment of PJI. In this review, the most common reasons for tigecycline use were renal failure and treatment of culture negative or polymicrobial infection, but other reasons included organism resistance and antimicrobial allergies. The rationale for tigecycline therapy was similar to another study in which patients were treated with tigecycline for osteomyelitis 17.

Comparable to other studies, the most common organism isolated in our study was *Staphylococcus aureus* 6. Interestingly, a large number of the infections (38%) were culture negative compared to the incidence of 7-11% quoted in the literature 1, 6. This is likely due to selection bias as tigecycline was chosen in many of our patients precisely because broad therapy was desired due to failure to isolate an organism. It is important to note that even though cultures remained negative in these patients they still met MSIS criteria for PJI. In one retrospective review of patients treated for culture negative PJI, the rate of treatment success was 69.2% 18. Similarly, in our subset of culture negative patients, the success rate was 57%.

In addition to a large number of culture negative infections, our study included seven patients with polymicrobial infections. Studies have suggested a higher risk of treatment failure with polymicrobial infection 19. Contrary to what we expected, patients in our study with monomicrobial infections had the worst outcomes with a success rate of only 33%. We wonder if these patients would have benefited from combination therapy of tigecycline plus another agent as some studies have suggested 16, 20.

When accounting for differences in surgical management, our success rates were lower than expected, but we attribute this partially to the medical and surgical complexity of the patient population. The majority of patients (54%) suffered co-morbidities including diabetes and kidney disease, and the average BMI was 35 mg/m^2^ which increases the risk for post-operative infection significantly 21. Of the 37 patients in our study, at least 22 (59%) had previously been treated for an infection in the joint under review. The majority of patients (51%) underwent two-stage revisions. It is estimated that the relapse rate for a patient receiving a two-stage revision for a hip arthroplasty is 4-16% and for a knee arthroplasty is 3-16% 22, but there are no good data on the failure rate of patients undergoing two-stage revision in the setting of prior PJI.

Only one patient experienced side effects severe enough to require discontinuation of tigecycline therapy. This patient developed elevated liver enzymes; therefore, treatment was discontinued early. This frequency of adverse effects is similar to other studies 17, 23. Overall, tigecycline was well tolerated, and patients received an average of 40 days of therapy.

There were several limitations to this study. It was a retrospective review and likely influenced by selection bias. It was performed at a single institution, which limits generalizability. The sample size was small, limiting the ability to draw significant statistical conclusions from the data. Because we required patients to receive tigecycline for greater than 75% of their treatment course, we were not able to assess many patients who were started on a standard of care regimen such as vancomycin and then switched to tigecycline after they developed an adverse reaction. These patients likely represent the majority of patients who would receive tigecycline in clinical practice. Finally, the follow-up for some of the patients was short.

As PJI is expected to become more common, it is important to continue to evaluate effective treatment regimens. Many patients with PJI have multiple co-morbidities making antimicrobial selection challenging. Resistant organisms and polymicrobial infections also pose a treatment dilemma. To date, our study is the largest case series describing the use of tigecycline for treatment of PJI. Our success rate was lower than expected, and we propose that tigecycline should be reserved for cases of PJI where all other treatment options have been exhausted. The International Consensus on Orthopedic Infections recently published in its proceedings that tigecycline should be considered an option in the treatment of multi-drug resistant gram positive infections or gram negative infections in combination with another antimicrobial 24. Our patients received tigecycline monotherapy. In the future, it will be important to study the use of tigecycline in combination with other agents such as rifampin, colistin, and fosfomycin as a means to improve treatment outcomes.

## Supplementary Material

Supplementary tables.

## Figures and Tables

**Table 1:** Demographics and Antimicrobial Details.

Sex	
Female	24
Male	13
**Co-Morbidities**	
Chronic Renal Failure	11
Diabetes	10
Immunosuppressed	4
Tigecycline Reason^*^	
Empiric/ Culture Negative	14
Renal failure	11
Polymicrobial Infection	7
Resistant Organism	5
Antimicrobial Allergy	3
Other	1
**Sequence in Episode**	
1^st^ in this episode, no prior courses of IV antimicrobial therapy	12
1^st^ in this episode, but prior courses of IV antimicrobial therapy	20
2^nd^ in this episode	5
**Number of Prior Courses of Antimicrobials**	
0	13
At least 1	17
At least 2	3
At least 3	2
Unknown	2

* Number of reasons is greater than total number of patients in study as some patients had several reasons for tigecycline selection.

**Table 2:** Treatment Details

Prosthesis Type	
THA	19
TKA	15
Combined TKA & THA	1
TSA	2
**Surgical Procedure**	
Debridement and Prosthesis Retention	5
Single Stage Revision	1
2 Stage Revision	19
2 + Stage Revision	9
Fusion	2
Non-operative Management	1
**Outcome**	
Success	16
Failure	21

THA, total hip arthroplasty; TKA, total knee arthroplasty; TSA, total shoulder arthroplasty.

**Table 3:** Success Rate by Surgical Procedure and Culture Results.

Surgical Procedure	Number of Successes/ Total	Success Rate (%)
Debridement and Prosthesis Retention	1/5	20%
Single Stage Revision	0/1	0%
2 Stage Revision	11/19	58%
2 + Stage Revision	3/9	33%
Fusion	1/2	50%
Non-operative Management	0/1	0%
**Culture Results**		
Polymicrobial Infection	3/7	43%
Monomicrobial Infection	5/15	33%
Culture Negative	8/14	57%

## Abbreviations

PJI: Prosthetic Joint Infection
IV: Intravenous
MRSA: Methicillin Resistant *Staphylococcus aureus*
VRE: Vancomycin resistant *Enterococcus*
MSIS: Musculoskeletal Infection Society
ICD: International Classification of Diseases
BMI: Body Mass Index
MSSA: Methicillin Susceptible *Staphylococcus aureus*
CoNS: coagulase-negative staphylococci
TKA: Total Knee Arthroplasty
THA: Total Hip Arthroplasty
TSA: Total Shoulder Arthroplasty
FDA: Food and Drug Administration
