# Supplementary material for: Clinical Experience with Tigecycline in the Treatment of Prosthetic Joint Infections
Source: J Bone Jt Infect. 2019 May 21;4(3):126–32. doi: 10.7150/jbji.34866 (PMC6536800; doi:10.7150/jbji.34866)
Supplement: Supplementary file 1 — Supplementary tables. [file jbjiv04p0126s1.pdf]

Table S1. Demographics and Antimicrobial Details

Patient	Age (years)	Sex	Weight (kg)	BMI (kg/m^2)	Co-Morbidities	Tigecycline Reason	Sequence in this Episode	# of Prior Antimicrobial Courses
**1**	66	M	121.9	39.5	none	empiric/ culture negative	1st but prior course	1
**2**	68	F	83.2	33.6	CRF (Stage 3)	*C difficile* colitis, rash on prior regimen (vancomycin and ceftazidime), empiric/ culture negative	2nd	1
**3**	60	F	100.1	39.1	none	*C difficile* colitis, prior CoNS and *E coli*, empiric/ culture negative	1st but prior course	1
**4**	70	M	78.3	25.5	CRF (Stage 3)	AKI on vancomycin and cefazolin, polymicrobial coverage	2nd	0
**5**	67	F	91.5	39.4	none	empiric/ culture negative	1st but prior course	1
**6**	57	M	183	Not available	DM, CRF (Stage 3)	targeted MRSA coverage	1st but prior course	1
**7**	54	F	121	41.9	none	AKI on vancomycin, allergy to daptomycin and penicillins, targeted MSSA coverage	2nd	0
**8**	65	F	85.9	37.0	DM	AKI, polymicrobial coverage	1st	0
**9**	47	M	135	Not available	DM, CRF (Stage 3)	CKD, targeted Strep coverage	2nd	0
**10**	64	F	89.8	Not available	Immunosuppressed	empiric/ culture negative (no ID note)	1st	unknown
**11**	57	M	95.2	Not available	DM	targeted *Cutibacterium acnes* coverage	1st	unknown
**12**	71	M	98	29.4	none	polymicrobial infection	1st	0
**13**	71	F	80.6	35.2	none	empiric/ culture negative	1st but prior course	at least 1
**14**	70	M	122.2	39.2	none	AKI with vancomycin and pip-tazo, targeted MRSA coverage	1st but prior course	1
**15**	61	M	123.8	37.0	CRF (Stage 3)	CKD, empiric/ culture negative	1st but prior course	at least 1
**16**	77	F	106.2	45.7	CRF (Stage 3)	CKD, empiric/ culture negative	1st but prior course	1
**17**	70	M	180.2	49.7	DM	concern about dosing vancomycin due to BMI, targeted CoNS coverage	1st but prior course	1
**18**	80	F	59	21.5	none	*C difficile* colitis, polymicrobial infection	1st but prior course	1
**19**	66	F	82	30.0	DM, CRF (Stage 3)	CKD, targeted MSSA	1st but prior course	1
**20**	65	F	52	18.5	DM, Immunosuppressed	history of infection with multi-drug resistant *E coli* as well as MSSA	1st but prior courses	at least 2
**21**	65	F	58	Not available	none	targeted *Streptococcus agalactiae* coverage	1st but prior courses	at least 2
**22**	53	F	88.5	Not available	none	desire to add antibacterial coverage when OR cultures grew *Candida*	1st but prior course	1
**23**	65	M	131	Not available	none	polymicrobial coverage	1st but prior course	at least 2
**24**	75	F	57	17.0	Immunosuppressed	polymicrobial coverage, resistant organisms	1st	0
**25**	74	F	103.6	41.8	none	targeted *Peptostreptococcus* coverage	1st	0
**26**	64	F	101	Not available	none	polymicrobial coverage	1st	0
**27**	88	F	63.4	26.4	CRF (Stage 4)	CKD, targeted *Enterobacter* coverage	1st	0
**28**	63	M	117.5	35.1	CRF (Stage 3)	CKD, empiric/ culture negative	1st	0
**29**	51	F	121.1	47.3	DM	vancomycin allergy, history of infection with *Enterococcus faecalis*, empiric/ culture negative	1st but prior course	1
**30**	68	F	124	51.1	DM	VRE coverage	1st but prior course	at least 1
**31**	53	M	148.0	45.6	CRF (Stage 3)	VRE with elevated daptomycin MIC	1st	0
**32**	59	M	66.0	19.7	Immunosuppressed	empiric/ culture negative	1st but prior course	1
**33**	66	F	72.8	26.7	none	history of infection with MRSA and VRE, empiric/ culture negative	1st but prior course	at least 3
**34**	65	F	82.5	34.4	none	desire to add antibacterial coverage when gram stain showed yeast	1st	0
**35**	59	F	99.0	38.7	none	MSSA with high vancomycin MIC	1st but prior course	at least 3
**36**	63	F	119.0	42.5	DM	targeted MRSA coverage	1st	0
**37**	66	F	135.0	Not available	none	AKI on vancomycin, empiric/ culture negative	2nd	1

M, male; F, female; CRF, chronic renal failure; CoNS, coagulase negative staphylococci; *E. coli*, *Escherichia coli*; DM, diabetes mellitus; AKI, acute kidney injury; ID, Infectious Diseases; CKD, chronic kidney disease; pip-tazo, piperacillin-tazobactam; BMI, body mass index; OR, operating room; MRSA, methicillin resistant *Staphylococcus aureus*; MSSA, methicillin susceptible *Staphylococcus aureus*; *C difficile*, *Clostridium difficile*; VRE, vancomycin resistant *Enterococcus*; MIC, minimal inhibitory concentration

Table S2. Characterization of Infections and Treatments

Patient	Prosthesis Type	Surgical Procedure	Pathogen	Resistance Profile	Duration (days)	Total antibiotic days	Total Time under Tigecycline (%)	Adverse Events	Outcome	Follow-up (months)
**1**	TKA	2 stage replacement	Culture negative	N/A	42	42	100	None	Success	28
**2**	THA	2+ stage replacement	Culture negative	N/A	32	42	76	None	Success	39
**3**	TKA	2 stage replacement	Gram stain with gpc in pairs, culture negative<br>Past cultures with CoNS, *E. coli*	N/A<br>*E. coli*- CIP	42	42	100	None	Success	24
**4**	TKA	D+PR	*Cutibacterium*, *Corynebacterium*	Not available	42	42	100	None	Failure within 29 months due to dislocation	29
**5**	THA	2 stage replacement	Culture negative	N/A	42	42	100	None	Success	24
**6**	TKA	2 stage replacement	MRSA	CIP, CLI, LVX, OX	42	42	100	None	Failure within 5 months	43
**7**	TKA	D+PR	MSSA	N/A	52	62	84	None	Failure within 5 months	15
**8**	TKA	2 stage replacement	*Cutibacterium*, *Streptococcus viridans*	Not available	42	42	100	None	Success	17
**9**	THA	2 stage replacement	*Streptococcus agalactiae*	No resistance	33	42	79	None	Success	45
**10**	TKA	2 stage replacement	Culture negative	N/A	42	42	100	None	Failure within 3 months	20
**11**	THA	2+ stage replacement	*Cutibacterium acnes*	Not available	42	42	100	None	Failure, details unknown	100 (by phone)
**12**	THA	2 stage replacement	*Actinomyces israelii*, MSSA, *Cutibacterium* species	No resistance	42	42	100	None	Success	13
**13**	THA	fusion	Culture negative	N/A	42	42	100	None	Success	24
**14**	THA	D+PR	MRSA<br>Past cultures with MRSA	CLI, OX	42	42	100	None	Success	13
**15**	THA	2 stage replacement	Culture negative	N/A	42	42	100	None	Success	16
**16**	THA	2 stage replacement	Culture negative<br>Past cultures with VRE	N/A<br>VRE- not available	42	42	100	None	Failure within 5 months	18
**17**	TKA	2+ stage replacement	CoNS	No resistance	42	42	100	None	Failure within 5 months	16
**18**	TKA	2+ stage replacement	MRSA, *Candida albicans* (fluconazole added)	CLI, OX	42	42	100	None	Failure within 12 months	15
**19**	TKA	fusion	MSSA	CLI	42	42	100	None	Failure within 3 months	42
**20**	THA	no surgery	No operative cultures<br>Past cultures with multi-drug resistant *E coli*, MSSA, *Proteus*, *Candida albicans*	N/A	28	28	100	None	Failure within 0 months, continued to drain	42

**21**	THA	2 stage replacement	*Streptococcus agalactiae*	No resistance	42	42	100	None	Success	18
**22**	THA	2 stage replacement	*C. lusitaniae* (fluconazole added)	N/A	42	42	100	None	Failure within 4 months	17
**23**	THA	2 stage replacement	*E. coli*, *Candida albicans*	AMP, GENT, SXT, CIP, LVX	42	42	100	None	Failure within 2 months	12
**24**	THA	2 stage replacement	*Enterococcus faecium*, *E. coli*, *Providencia stuartii*	*Enterococcus*: VAN, AMP<br>*E. coli*: CFZ, CIP, AMI<br>*Providencia*: CIP	42	42	100	None	Failure within 15 months	15
**25**	TSA	2 stage replacement	*Peptostreptococcus* species	Not performed	42	42	100	None	Success	21
**26**	THA	2 stage replacement	*Staphylococcus lugdunensis*, multiple gram positives on gram stain	No resistance	42	42	100	None	Success	101
**27**	TKA	D+PR	*Enterobacter cloacae* complex	Augmentin only	28	28	100	Elevated AST & ALT (stopped early)	Failure within 39 months	38
**28**	TKA	2+ stage replacement	Culture negative	N/A	42	42	100	None	Failure within 2 months	40
**29**	THA	2+ stage replacement	Culture negative<br>Past cultures with *Enterococcus faecalis*	N/A<br>*E. faecalis*- no resistance	42	42	100	None	Failure within 5 months	17
**30**	TKA	2 stage replacement	*E. faecium*	VAN, AMP	42	42	100	None	Success	60
**31**	TKA	2+ stage replacement	*E. faecium*	VAN, DAP	42	42	100	None	Failure within 3 months	29
**32**	TSA	2+ stage replacement	Culture negative	N/A	42	42	100	None	Success	31
**33**	TKA	2+ stage replacement	Culture negative<br>Past cultures with MRSA and VRE	N/A<br>MRSA and VRE- not available	42	42	100	None	Success	52
**34**	THA	2 stage replacement	Gram stain with budding yeast, culture negative (fluconazole added)	N/A	42	42	100	None	Failure within 12 months	36
**35**	THA, TKA	1 stage replacement	MSSA	VAN	42	56	75	None	Failure while still on IV antimicrobials	20
**36**	THA	D+PR	MRSA	OX	42	42	100	None	Failure within 9 months	50
**37**	THA	2 stage replacement	Culture negative	N/A	33	42	79	None	Failure within 6 months due to dislocation	106

TKA, total knee arthroplasty; THA, total hip arthroplasty; TSA, total should arthroplasty; N/A, not applicable; gpc, gram positive cocci; *E. coli*, *Escherichia coli*; D+PR, debridement plus prosthesis retention; MRSA, methicillin resistant *Staphylococcus aureus*; MSSA, methicillin susceptible *Staphylococcus aureus*; CIP, ciprofloxacin; CLI, clindamycin; LVX, levofloxacin; OX, oxacillin; VRE,

vancomycin resistant *Enterococcus*; CoNS, coagulase-negative staphylococci; AMP, ampicillin; GENT, gentamicin; SXT, trimethoprim-sulfamethoxazole; VAN, vancomycin; CFZ, ceftazidime; AMI, amikacin; AST, aspartate aminotransferase; ALT, alanine aminotransferase; *E. faecalis*, *Enterococcus faecalis*; DAP, daptomycin
